# Activating Transcription Factor 4 (ATF4) modulates Rho GTPase levels and function via regulation of RhoGDIα

**DOI:** 10.1038/srep36952

**Published:** 2016-11-14

**Authors:** Silvia Pasini, Jin Liu, Carlo Corona, Eugenie Peze-Heidsieck, Michael Shelanski, Lloyd A. Greene

**Affiliations:** 1 Department of Pathology & Cell Biology and Taub Institute for Research on Alzheimer’s Disease and the Aging Brain, Columbia University Medical Center, New York, NY 10032, USA

## Abstract

In earlier studies, we showed that ATF4 down-regulation affects post-synaptic development and dendritic spine morphology in neurons through increased turnover of the Rho GTPase Cell Division Cycle 42 (Cdc42) protein. Here, we find that ATF4 down-regulation in both hippocampal and cortical neuron cultures reduces protein and message levels of RhoGDIα, a stabilizer of the Rho GTPases including Cdc42. This effect is rescued by an shATF4-resistant active form of ATF4, but not by a mutant that lacks transcriptional activity. This is, at least in part, due to the fact that Arhgdia, the gene encoding RhoGDIα, is a direct transcriptional target of ATF4 as is shown in ChIP assays. This pathway is not restricted to neurons. This is seen in an impairment of cell migration on ATF4 reduction in non-neuronal cells. In conclusion, we have identified a new cellular pathway in which ATF4 regulates the expression of RhoGDIα that in turn affects Rho GTPase protein levels, and thereby, controls cellular functions as diverse as memory and cell motility.

Activating Transcription Factor 4 (ATF4) is a ubiquitously expressed member of the ATF/CREB transcription factor family of basic leucine zipper domain proteins that is involved in a wide range of activities and biological functions^1^. Over the past decade or so, major emphasis has been placed on the role of ATF4 in responding to a variety of cellular stresses such as amino acid deprivation, oxidative stress, endoplasmic reticulum stress, and mitochondrial stress^2^,^3^,^4^,^5^,^6^. Such stresses lead to ATF4 elevation, which in turn activates pathways leading to either cell protection or destruction^7^,^8^.

Aside from its role in the stress response, additional studies have implicated ATF4 in brain function as a pivotal regulator of synaptic plasticity and memory^9^,^10^,^11^,^12^,^13^,^14^. However, the ATF4 targets that mediate its actions in the nervous system have not been defined. To understand the role of basal ATF4 expression in unstressed cells and in particular in neurons, we have utilized the strategy of knocking down ATF4 *in vitro* and *in vivo* with shRNAs and assessing the consequences at both the cellular and behavioral levels. In cultured neurons and in the intact hippocampus, down-regulation of ATF4 leads to a reduction in the density of dendritic mushroom spines, which appear to play important roles in learning and memory^15^. In culture, loss of spines caused by ATF4 down-regulation is associated with a decrease in density of excitatory glutamatergic synapses^15^. Moreover, knockdown of ATF4 in the hippocampus impairs long-term potentiation (LTP) as well as long-term depression (LTD) and leads to deficits in spatial memory and behavioral flexibility^16^.

Our studies on the mechanisms underlying ATF4′s effects on dendritic spines and excitatory synapses revealed that depletion of basal ATF4 in cortical and hippocampal neurons elevates turnover of the Rho GTPase Cell Division Cycle 42 (Cdc42) protein without affecting its synthesis, thereby lowering total protein expression by 40–60%^15^. ATF4 knockdown also results in a corresponding decrease in levels of active GTP-bound Cdc42. Cdc42 has been characterized as a key regulator of actin cytoskeleton remodeling both within and outside of the nervous system^17^,^18^,^19^,^20^. In neurons, Cdc42 is necessary for spine and synapse formation^21^,^22^, raising the idea that at least some of the responses to ATF4 knockdown are mediated by Cdc42 depletion. In support of this, Cdc42 knockdown qualitatively reproduces the effects of ATF4 knockdown on densities of mushroom spines and of an excitatory synapse marker^15^. Also of note, knockdown of ATF4 additionally leads to a decreased expression of total and active forms of the Rho GTPase family member RhoA, although this effect does not appear to contribute to loss of spines or excitatory synapses^15^.

Although ATF4 knockdown leads to depletion of Cdc42 by increasing its turnover this change is not accompanied by a change in Cdc42 mRNA levels^15^. Nevertheless, the ability of ATF4 to regulate Cdc42 levels appears to require ATF4′s transcriptional activity. The effect of ATF4 knockdown on Cdc42 expression is rescued by over-expression of wild-type ATF4, but is not rescued by comparable over-expression of a form of ATF4 mutated to eliminate its transcriptional activity^15^. This could be explained by ATF4-mediated transcriptional regulation of another gene(s) that in turn affects Cdc42 stability and turnover.

One candidate for this “missing link” is the gene Arhgdia, which encodes RhoGDIα, a protein that binds and stabilizes cytoplasmic Rho GTPase family members including Cdc42^23^,^24^. We find that basal ATF4 levels are required for normal expression of RhoGDIα, and that Arhgdia is a direct transcriptional target of ATF4. Finally, we report that the ATF4-RhoGDIα-Cdc42 pathway is present in non-neuronal cells as well as neurons and that ATF4 depletion leads to impaired cell migration.

## Results

### ATF4 down-regulation decreases RhoGDIα protein levels in cultured rat hippocampal and cortical neurons

We previously reported that ATF4 regulates Cdc42 protein expression by affecting its stability and not its mRNA levels^15^. Because ATF4 is a transcription factor, this suggested an effect on another component(s) that impinge(s) on Cdc42 turnover. Because Rho GTPase proteins are degraded by the proteasome^25^, we first assessed whether ATF4 knockdown affects the expression of E3 ubiquitin ligases. We carried out the knockdown as previously described^15^ by infecting cultured hippocampal or cortical neurons at 5 *DIV* with lenti-shATF4 or lenti-shCTRL. In response to lenti-shATF4, ATF4 mRNA was downregulated by over 80% at 4 days after infection of either neuron type and remained so for at least 12 days (Supplementary Fig. 1a,b). There is no identified E3 ubiquitin ligase specific for Cdc42, but Smurf1 has been shown to mediate degradation of RhoA^26^, the levels of which also decrease with ATF4 knockdown^15^. However, under our ATF4 knockdown conditions we did not observe significant changes in Smurf1 mRNA (Supplementary Fig. 1c,d), or in the mRNA levels of either the E3 ubiquitin ligase UBE3A (Supplementary Fig. 1e), which targets two RhoA GEFs for proteasome degradation^27^,^28^, or the ubiquitin c-terminal hydrolase, UCH-L1, which may be directly involved in the proteasome degradation of Rho GTPases^29^ (Supplementary Fig. 1f).

**Figure 1:**
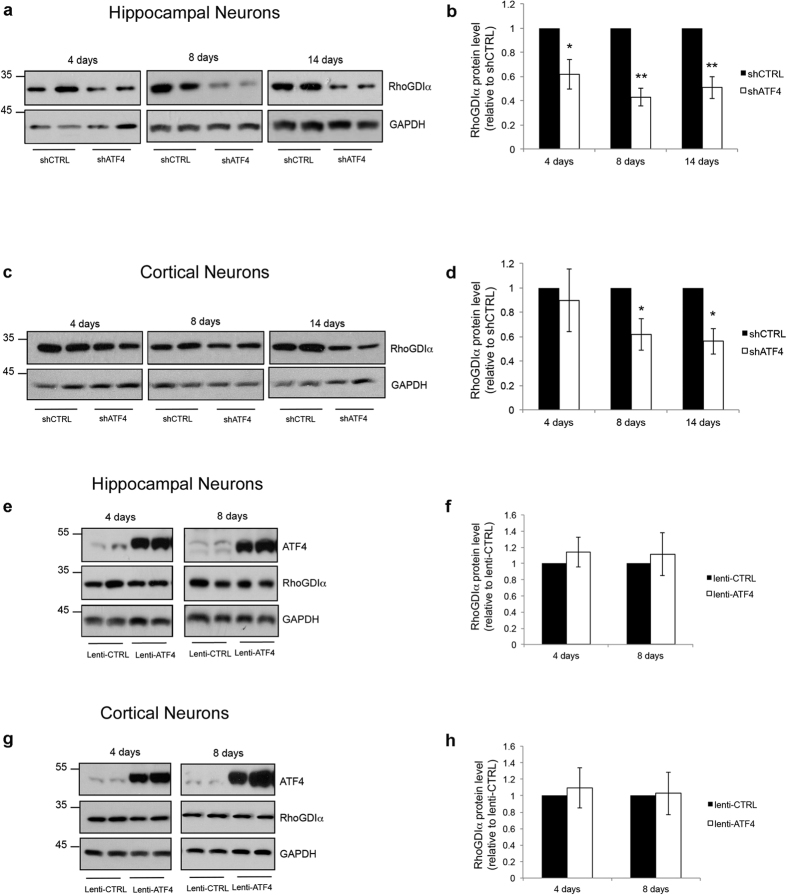
ATF4 knockdown decreases RhoGDIα protein levels in cultures of rat hippocampal and cortical neurons. (**a,c**) Cropped representative Western immunoblots showing a time course of the effect of ATF4 downregulation on neuronal RhoGDIα protein levels. Cultures of hippocampal (**a**) and cortical (**c**) neurons were infected with lenti-shCTRL or lenti-shATF4 at 5 *DIV* and total cell lysates were collected at the indicated time points after the infection and analyzed by Western immunoblotting. (**b,d**) Quantification of RhoGDIα protein levels at the indicated time points relative to control in hippocampal neurons (**b**) and cortical neurons (**d**). Data are expressed as mean ± sem of independent experiments (**b**, 4 days n = 5, 8–14 days n = 8; **d**, n = 3) (*p < 0.05, **p < 0.001). (**e,g**) Cropped representative Western immunoblots showing a time course of the effect of ATF4 overexpression on RhoGDIα protein levels. Cultures of hippocampal (**e**) and cortical (**g**) neurons were infected with lenti-CTRL or lenti-ATF4 at 5 *DIV* and total cell lysates were collected at the indicated time points after the infection and analyzed by Western immunoblotting. (**f,h**) Quantification of RhoGDIα protein levels at the indicated time points relative to control in hippocampal neurons (**f**) and cortical neurons (**h**). Data are expressed as mean ± sem of 3 independent experiments. Full-length Western immunoblots are shown in Supplementary Figure 6.

Next, we focused on the Rho GDP dissociation inhibitor-alpha (RhoGDIα), the product of the Arhgdia gene. Although usually studied as a regulator of Rho GTPase activity and localization, RhoGDIα has also been reported to bind and stabilize members of the Rho family including Cdc42^30^. To determine whether ATF4 might regulate RhoGDIα expression, we knocked down ATF4 in hippocampal and cortical cultures and assessed RhoGDIα protein at different time points. In hippocampal neurons, ATF4 depletion significantly reduced RhoGDIα protein expression at 4 days and this decrease was maintained for at least 14 days (Fig. 1a,b). ATF4 knockdown also diminished RhoGDIα protein levels in cortical neuron cultures, although this effect was not significant until day 8 (Fig. 1c,d).

Overexpression of ATF4 (using a previously described lentiviral construct^15^) did not significantly alter RhoGDIα protein levels in either neuron type (Fig. 1e–h). This is consistent with our past findings that in contrast to the effects of ATF4 knockdown on Cdc42 levels, densities of mushroom spines and excitatory synaptic markers, ATF4 over-expression does not affect these parameters^15^.

### RhoGDIα knockdown reduces levels of total and active forms of Cdc42, as well as of RhoA, and Rac1, in cultured hippocampal and cortical neurons

**Figure 2:**
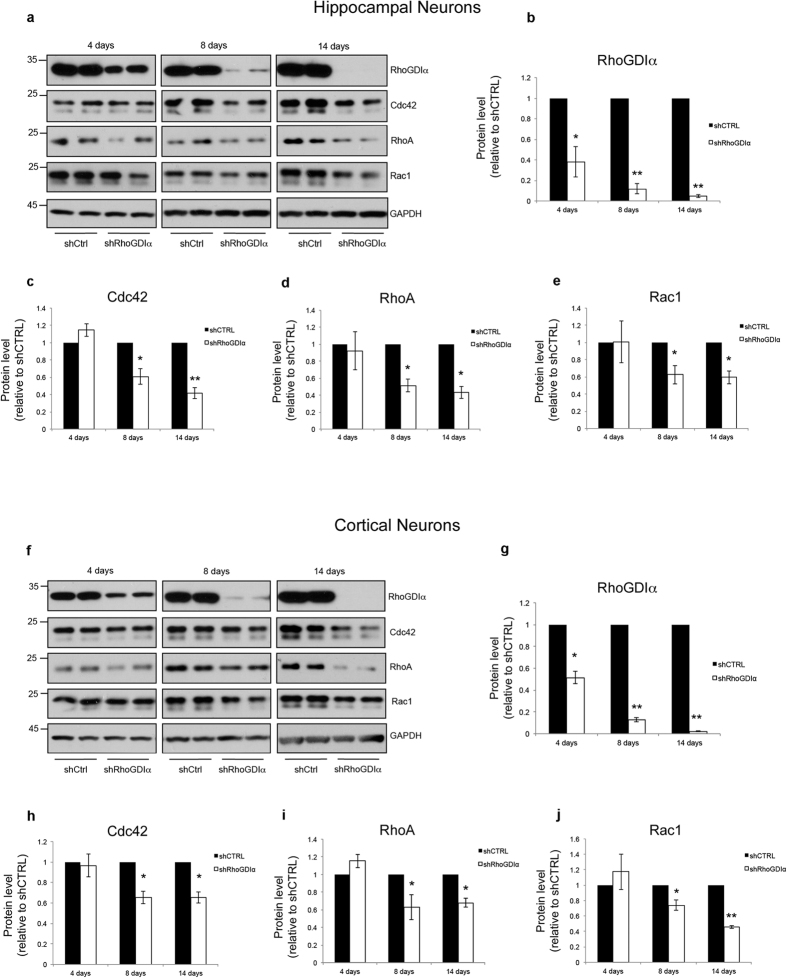
Long-term RhoGDIα knockdown in cultures of hippocampal and cortical neurons reduces the total levels of Cdc42, RhoA, and Rac1 proteins. (**a**) Cropped representative Western immunoblots showing a time course of the effect of RhoGDIα knockdown on Rho GTPase protein levels in hippocampal neurons. Cultured neurons were infected with lenti-shCTRL or lenti-shRhoGDIα at 5 *DIV* and total cell lysates were collected at the indicated time points after infection and analyzed by Western immunoblotting. (**b–e**) Quantification of RhoGDIα (**b**), Cdc42 (**c**), RhoA (**d**), and Rac1 (**e**) protein levels at the indicated time points relative to control. Data are expressed as mean ± sem of independent experiments (4 days n = 4, 8 days n = 6, 14 days n = 5) (*p < 0.05, **p < 0.001). (**f**) Cropped representative Western immunoblots showing a time course of the effect of RhoGDIα knockdown on Rho GTPase protein levels in primary cortical neurons. Cultured neurons were infected with lenti-shCTRL or lenti-shRhoGDIα at 5 *DIV* and total cell lysates were collected at the indicated time points after infection and analyzed by Western immunoblotting. (**g–j**) Quantification of RhoGDIα (**g**), Cdc42 (**h**), RhoA (**i**), and Rac1 (**j**) protein levels at the indicated time points relative to control. Data are expressed as mean ± sem of independent experiments (4 days n = 3, 8 days n = 6, 14 days n = 4) (*p < 0.05, **p < 0.001). Full-length Western immunoblots are shown in Supplementary Figure 7.

We previously found that ATF4 knockdown in neurons leads to a significant reduction in the levels of total and activated Cdc42 (as well as of RhoA)^15^. Because ATF4 knockdown reduces neuronal RhoGDIα, and because RhoGDIα knockdown diminishes Rho family GTPase protein levels in different cell types, including Hela, fibroblast, breast epithelial, melanoma, and endothelial cells^30^, we asked whether RhoGDIα depletion would mimic the effect of ATF4 knockdown on Rho family protein levels in neurons. To do this, we generated lentiviruses carrying a shRNA sequence that specifically targets RhoGDIα mRNA^31^, and used it to infect primary hippocampal and cortical neuron cultures. In both neuron types, there was a 50–60% reduction in RhoGDIα protein by 4 days after infection; this reduction progressed over time so that the knockdown was 90% or greater by 8 days after infection (Fig. 2a,b,f,g). When we assessed the total levels of the three Rho GTPases, Cdc42, RhoA, and Rac1, we found that they were all significantly reduced by 8 days following RhoGDIα knockdown (Fig. 2a,c–e,f,h–j). To rule out the possibility that the results were due to an off target effect of the shRNA sequence, we repeated the experiments using a second RhoGDIα shRNA (shRhoGDIα#2)^32^. The results were similar to those with the first shRNA sequence, confirming that RhoGDIα downregulation reduces Cdc42, RhoA, and Rac1 in both hippocampal and cortical neurons (Supplementary Fig. 2). Our findings thus corroborate in neurons the observation in non-neuronal cells that depletion of RhoGDIα leads to reduction of Rho GTPases^30^ and support the hypothesis that ATF4 regulates neuronal Cdc42 and RhoA levels via its effects on RhoGDIα protein expression.

Our results show that knockdown of ATF4 reduces expression of RhoGDIα and that knockdown of RhoGDIα reduces neuronal expression of Rho family GTPases. Since knockdown of ATF4 in hippocampal neurons leads to a reduction of both RhoGDIα and Cdc42 proteins, the data strongly supports an ATF4- RhoGDIα-RhoGTPase pathway (Supplementary Fig. 3).

**Figure 3:**
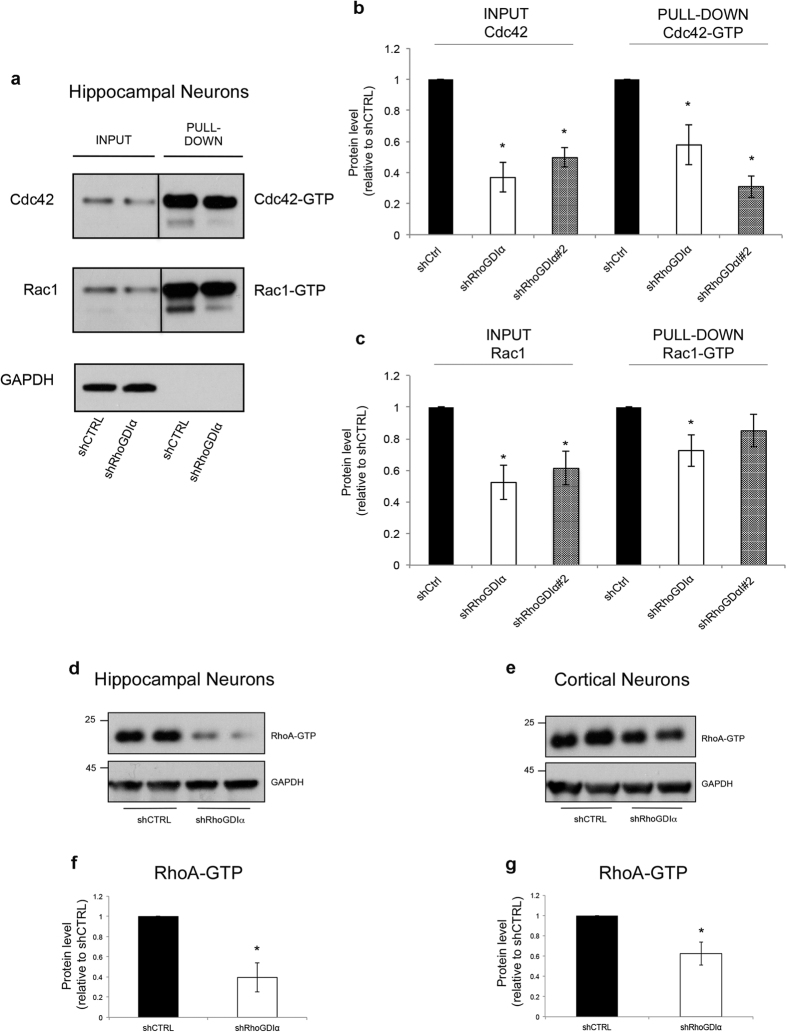
RhoGDIα knockdown in cultures of hippocampal and cortical neurons reduces the active forms of the Rho GTPase Cdc42, Rac1, and RhoA. (**a**) Cropped representative Western immunoblots showing the effect of RhoGDIα knockdown on Cdc42-GTP and Rac1-GTP protein levels. Cultured hippocampal neurons were infected at 5 *DIV* with lenti-shCTRL or lenti-shRhoGDIα for 10 days and protein lysates were collected and subjected to immunoprecipitation by using PAK-GST beads that bind to the GTP-bound Cdc42 and Rac1. Cdc42-GTP and Rac1-GTP were detected by Western immunoblotting with anti-Cdc42 and anti-Rac1 antibodies. (**b**) Quantification of total Ccd42 (INPUT) and Cdc42-GTP (PULL-DOWN) levels relative to control. (**c**) Quantification of total Rac1 (INPUT) and Rac1-GTP (PULL-DOWN) levels relative to control. Data are expressed as mean ± sem of 4 independent experiments (*p < 0.05). (**d,e**) Cropped representative Western immunoblots showing the effect of RhoGDIα knockdown on RhoA-GTP protein levels in cultured hippocampal (**d**) and cortical (**e**) neurons. Cultured hippocampal and cortical neurons were infected at 5 *DIV* with lenti-shCTRL or lenti-shRhoGDIα and total cell lysates were collected 10 days after infection and analyzed by Western immunoblotting using an antibody that binds specifically to RhoA-GTP. (**f,g**) Quantification of RhoA-GTP levels relative to control after RhoGDIα knockdown in cultures of hippocampal (**f**) and cortical (**e**) neurons. Data are expressed as mean ± sem of 3 independent experiments (*p < 0.05). Full-length Western immunoblots are shown in Supplementary Figure 7.

In addition to diminishing total levels of Cdc42 and RhoA, we previously found that ATF4 knockdown leads to a comparable loss of the active, GTP-bound forms of these proteins^15^. To determine whether this might also be a consequence of the effect of ATF4 on RhoGDIα expression, we used lenti-shRhoGDIα and lenti-shRhoGDIα#2 to deplete RhoGDIα in cortical and/or hippocampal neuron cultures and assessed levels of GTP-bound Rho proteins. Using PAK-GST beads that specifically bind Cdc42-GTP and Rac1-GTP, we observed that RhoGDIα knockdown for 10 days significantly reduces the activated forms of both proteins (in the case of shRhoGDIα#2 the decrease in Rac1-GTP did not reach significance) (Fig. 3a–c). We also measured RhoA-GTP levels by Western Immunoblotting using an antibody that specifically recognizes the active form of the protein. Here too, knockdown of RhoGDIα led to loss of RhoA-GTP (Fig. 3d–g, Supplementary Fig. 4a–d). These findings support our hypothesis that ATF4 affects levels of Cdc42-GTP and RhoA-GTP through its actions on RhoGDIα expression and suggest that the reduction in Rho GTPase activity likely follows from the loss of the corresponding total protein.

### Transcriptional regulation of RhoGDIα by ATF4

**Figure 4:**
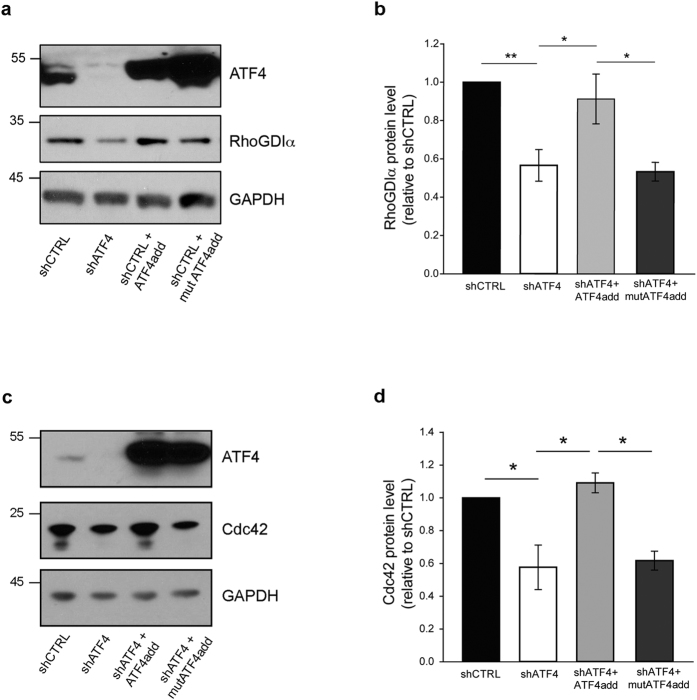
Regulation of RhoGDIα and Cdc42 by ATF4 requires ATF4′s transcriptional activity. (**a**) Cropped representative Western immunoblots showing that regulation of RhoGDIα by ATF4 requires ATF4′s transcriptional activity. Cultured hippocampal neurons were infected at 5 *DIV* with the indicated lenti-shRNAs. 10 days after infection protein lysates were collected and subjected to Western immunoblotting. (**b**) Quantification of RhoGDIα protein levels relative to control condition. Data are expressed as mean ± sem of 6 independent experiments (*p < 0.05, **p < 0.001). (**c**) Cropped representative Western immunoblots showing that regulation of Cdc42 by ATF4 requires ATF4′s transcriptional activity. Cultured hippocampal neurons were infected at 5 *DIV* with the indicated lenti-shRNAs. 10 days after infection protein lysates were collected and subjected to Western immunoblotting. (**d**) Quantification of Cdc42 protein levels relative to control condition. Data are expressed as mean ± sem of 3 independent experiments (*p ≤ 0.05). Full-length Western immunoblots are shown in Supplementary Figure 7.

We have previously performed rescue experiments in which we used lentiviral delivery of a modified form of ATF4 cDNA (ATF4add) that encodes the same amino acid sequence as wild-type ATF4, but that is not recognized by shATF4^15^,^16^. When co-infected into hippocampal neurons, ATF4add reversed the reduction of RhoGDIα protein caused by shATF4, thus ruling out possible off-target shRNA effects (Fig. 4a,b). As in the case of wild-type ATF4 over-expression (Fig. 1e,f), infection with the rescue construct did not further increase RhoGDIα levels above those under the control conditions (Fig. 4a,b).

To test whether the actions of ATF4 require its transcriptional activity, we have generated an additional ATF4 construct (mutATF4add) that encodes a form of ATF4 that is not recognized by shATF4 and additionally does not bind DNA and is transcriptionally inactive, and that unlike ATF4add, does not rescue the effects of ATF4 knockdown on spine or synapse formation or on mEPSC frequency and amplitude^15^,^16^. In contrast to transcriptionally active ATF4add, mutATF4add did not reverse the decreases in RhoGDIα or Cdc42 protein levels caused by shATF4 (Fig. 4a–d). Together these results suggest that RhoGDIα and Cdc42 reductions that occur in response to shATF4 are specifically caused by a decline in ATF4 levels, and that the regulation of RhoGDIα and Cdc42 by ATF4 requires its transcriptional activity.

**Figure 5:**
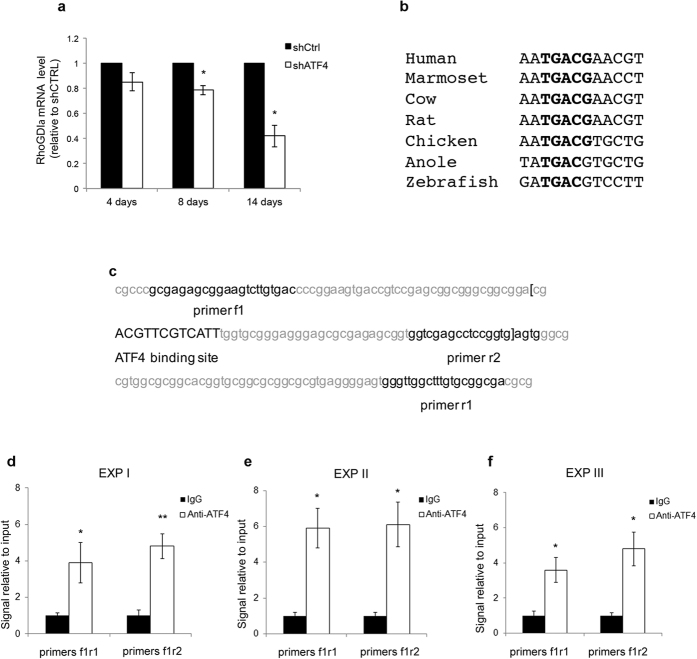
Transcriptional control of RhoGDIα by ATF4. (**a**) Real time PCR showing RhoGDIα mRNA levels at different time points after ATF4 knockdown. Total RNA was extracted from cultured hippocampal neurons at the indicated time points and subjected to quantitative real time PCR to detect RhoGDIα message levels. Data are expressed as mean ± sem of 3 independent experiments (*p < 0.05). (**b**) Analysis of the RhoGDIα/Arhgdia 5′ promoter region using the ConTra v2 webserver and the TRANSFAC position weight matrix. A predicted ATF4 binding site in the Arhgdia transcription start site region (bold uppercases) is conserved between species. (**c**) RhoGDIα/Arhgdia rat gene sequence containing the predicted ATF4 binding site (bold upper cases) in the noncoding region of exon 1. Bold lowercases represent the primer sequences used in the ChIP assays. Brackets show exonal sequence. (**d–f**) ChIP assays indicate an enrichment of ATF4 protein binding in the region of the rat RhoGDIα/Arhgdia gene containing a putative ATF4 binding site. Primary cortical neurons were cultured for 10 days with (**d**) or without (**e,f**) shCTRL lentivirus infection at *DIV* 5 followed by ChIP Assay. After crosslinking, the chromatin was sheared and immunoprecipitated using antibody against ATF4 or rabbit immunoglobulin G (IgG) isotype as control. The genomic DNA was purified and subjected to quantitative PCR using two different primer sets designed to amplify the predicted ATF4 binding site in the Arhgdia gene. Data are expressed as fold change relative to control signal (Rabbit IgG), after normalization to input. Each graph (**d–f**) represents an individual experiment performed in triplicate. Data are expressed as mean ± sem of triplicates (*p < 0.05, **p < 0.001).

Given the requirement for ATF4 transcriptional activity in the regulation of RhoGDIα protein expression, we asked whether this was due to an effect on RhoGDIα transcript levels. Primary hippocampal cultures were infected at 5 *DIV* with shATF4 or shCTRL and RhoGDIα mRNA levels were analyzed at various times thereafter by real-time PCR. This revealed that ATF4 knockdown causes a progressive decrease in RhoGDIα mRNA levels, with a 20% reduction at 8 days and a 60% loss by 14 days (Fig. 5a).

To assess whether RhoGDIα is a direct transcriptional target of ATF4, we carried out an analysis of the Arhgdia transcription start site region using the ConTra v2 webserver^33^ and the TRANSFAC position weight matrix. This revealed a putative ATF4 DNA binding site (AATGACGAACGT) that was conserved from rat to human in the noncoding region of exon 1 (Fig. 5b). The consensus sequence ATGACGT was also present in the chicken, anole and zebrafish Arhgdia 5′ UTR (Fig. 5b).

We verified this *in silico* prediction using chromatin immunoprecipitation (ChIP) assays. Cortical neurons were maintained for 10 days *in vitro*, with or without shCTRL lentivirus infection at *DIV 5*, followed by ChIP assays using anti-ATF4 or control IgG. Real time PCR was performed on the immunoprecipitated chromatin using two different sets of primer pairs designed to amplify the putative regions of the RhoGDIα/Arhgdia start site that span the predicted ATF4 binding site (Fig. 5c). The results showed a consistent 4–5 fold enrichment signal with ATF4 antibody compared to a control IgG both in non-infected neurons (Fig. 5d,e) and shCTRL-infected ones (Fig. 5f). These findings support the idea that the Arhgdia gene is a direct transcriptional target of ATF4.

### The ATF4-RhoGDIα-Cdc42 pathway is present in non-neuronal cells

**Figure 6:**
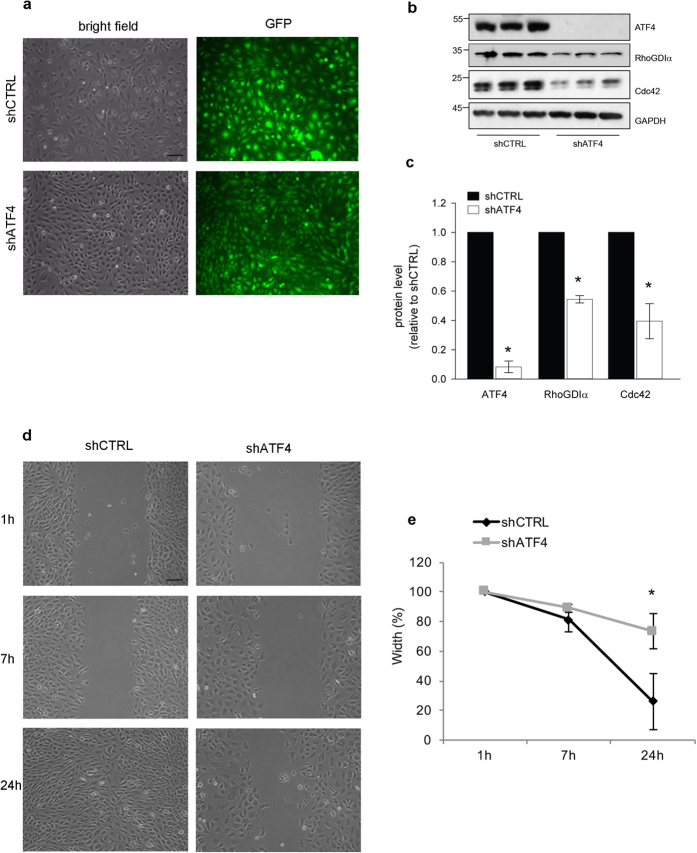
The ATF4-RhoGDIα-Cdc42 pathway is not neuronal specific. (**a**) Representative images of NRK cells subjected to a double infection (3 days apart) with either lenti-shCTRL or lenti-shATF4. Images were taken both in bright field (total cell) and green channel (infected cells) 10 days after the first infection. Scale bar 50 μm. (**b**) Cropped representative Western immunoblots showing the effect of ATF4 knockdown on RhoGDIα and Cdc42 protein levels in NRK cells. Full-length Western immunoblots are shown in Supplementary Figure 6. NRK cells were subjected to a double infection (3 days apart) with either lenti-shCTRL or lenti-shATF4. Total protein lysates were collected 10 days after the first infection for Western immunoblotting. (**c**) Quantification of ATF4, RhoGDIα, and Cdc42 protein levels in shATF4-infected cells compared with shCTRL-infected cells. Data represent the mean ± sem of 3 independent experiments (*p < 0.05). (**d**) Representative images of the response of NRK cells in a scratch assay with or without knockdown of ATF4. NRK cells were subjected to double infection (3 days apart) with either lenti-shCTRL or lenti-shATF4. 10 days after the first infection, monolayers of NRK cells were scratched with a 20 μl pipette tip and the wound width was measured at 1 h (time 0), 7 h, and 24 h after the scratch. Scale Bar 50 μm. (**e**) Quantification of scratch assays. Width closure (%) was measured over time and normalized to the width at 1 h (time 0). Data are expressed as mean ± sem of 3 independent experiments. Significant differences in the wound closure were observed at the 24 h time point (*p < 0.05).

Because ATF4, Cdc42 and RhoGDIα appear to be ubiquitously expressed^24^ we sought to determine whether the ATF4-RhoGDIα-Cdc42 pathway is present and physiologically relevant in other cell types in addition to neurons. To address this, we knocked down ATF4 in NRK cells (a line derived from rat kidney epithelium^34^) and Melan-a cells (an immortalized line of pigmented melanocytes, derived from epidermal melanoblasts of inbred C57BL mice^35^). The cultured cells were double infected (3 days apart) with either lenti-shCTRL or lenti-shATF4, previously demonstrated to work both in rat^15^ and mouse^16^. Both cell types underwent infection with the lentiviral constructs, with the efficiency (as judged by GFP expression) higher in NRK (Fig. 6a) than in Melan-a cells (Supplementary Fig. 5a). 10 days after the first infection with shATF4 NRK cells showed more than a 90% reduction (Fig. 6b,c), while Melan-a cells showed a 50% reduction in ATF4 protein levels (Supplementary Fig. 5b,c) compared to controls. Under these conditions of ATF4 knockdown, the levels of RhoGDIα and Cdc42 protein were lower in both cell types compared to controls. In NRK cells RhoGDIα expression was significantly decreased by 46% and Cdc42 levels by 62% (Fig. 6c), while in Melan-a cells RhoGDIα expression was decreased by 49% and Cdc42 significantly decreased by 67% (Supplementary Fig. 5c). Together these results suggest that, as in neurons, in non-neuronal cells diminished ATF4 expression leads to decreased expression of RhoGDIα which in turn leads to loss of Cdc42.

Because Rho GTPases, in particular Cdc42, regulate cell migration in many different cell types^36^,^37^, we performed scratch assays with NRK cells to measure their mobility with and without ATF4 knockdown. 10 days after a double infection with either shCTRL or shATF4, scratches were generated in the cell monolayer and the cultures were imaged 1 h, 7 h and 24 h later to assess the extent to which the cells filled in the gap (Fig. 6d,e). Cells in cultures infected with shCTRL migrated into the gap and nearly closed it (80%) by 24 h. In contrast, shATF4-infected cells showed considerably less migration into the gap with only a 20% closure by 24 h (Fig. 6d,e). These findings suggest that the pathway involving ATF4, RhoGDIα, and Cdc42 is conserved among different cell types and that it regulates important cellular functions including migration.

## Discussion

The studies presented here identify a new cellular pathway controlled by ATF4. In this pathway, a reduction in ATF4 reduces the expression of both RhoGDIα mRNA and protein. We also show that the ATF4-RhoGDIα-Cdc42 pathway is conserved in other cell types such as melanocytes and kidney epithelial cells and that reduction of ATF4 in such cells has physiologic consequences that reflect decreased RhoGDIα expression.

RhoGDIα is the most abundant and best characterized member of the RhoGDI family^38^, which also includes RhoGDIβ and RhoGDIγ. The former is mainly expressed in hematopoietic tissues^39^, while the latter is expressed in lung, kidney, testis, pancreas and in brain^40^,^41^,^42^. In contrast to effects on RhoGDIα mRNA, down-regulation of ATF4 in neuronal cultures did not alter transcript levels of RhoGDIγ (data not shown). Thus, it seems likely that RhoGDIα is the main member of the family affected by ATF4 knockdown in our studies.

An important role of RhoGDIα is to control cellular expression of Rho GTPase proteins including Cdc42, RhoA, and Rac1. Newly synthetized Rho GTPases are subject to prenylation, which increases their affinity for membranes, but makes them susceptible to proteasomal degradation when in the cytosol^30^,^43^,^44^. By binding cytosolic Rho GTPases, RhoGDIα stabilizes them and maintains them in an inactive pool that is available, when needed, to shuttle to the membrane for activation and transduction of signaling pathways^24^,^30^,^45^. Consistent with the finding that RhoGDIα regulates Rho GTPase stability in non-neuronal cells, we found that RhoGDIα knockdown led to significantly reduced levels of total and active forms of Cdc42, RhoA, and Rac1 in both hippocampal and cortical neurons.

We have reported that ATF4 knockdown leads to a reduction of total and active forms of Cdc42 in neurons and that this is due to enhanced Cdc42 protein turnover^15^. We have also shown that ATF4 depletion in neurons causes a reduction in mushroom spine density and of excitatory synapses and that these events are due, at least in part, to reduction of Cdc42 levels. The findings that ATF4 regulates RhoGDIα in neurons and that RhoGDIα knockown reduces Cdc42 expression support the view that RhoGDIα mediates the effects of ATF4 on Cdc42 and therefore on mushroom spines and excitatory synapses. We have additionally reported that ATF4 knockdown or knockout in hippocampal neurons interferes with excitatory neurotransmission and with LTP and LTD and leads to long-term deficits in spatial memory and behavioral flexibility^16^. Given the roles of Cdc42 in such events^46^, it is reasonable to postulate that these deficits are also mediated in part by a reduction in RhoGDIα.

In our initial studies, ATF4 knockdown led to reduction of total and active forms of RhoA as well as of Cdc42^15^. This is consistent with previous findings that RhoGDIα affects the stability of RhoA and with our observation here that RhoGDIα knockdown reduces neuronal RhoA levels. However, we also found that ATF4 knockdown did not affect Rac1 expression, which contrasts with the present observation that knockdown of RhoGDIα significantly reduces total and active forms of Rac1. This apparent distinction between the effects of ATF4 and RhoGDIα knockdown on Rac1 may be due to the greater reduction of RhoGDIα expression achieved with shRhoGDIα (>90%) as compared with shATF4 (40–60%). Previous studies indicate that total RhoGDIα protein levels appear to be equal to the sums of the levels of Cdc42, RhoA, and Rac1 and that the three Rho GTPases compete with one another for association with (and therefore stabililzation by) RhoGDIα^30^,^47^,^48^. Thus, one explanation for our findings would be that in neurons, RhoGDIα may have a stronger affinity for Rac1 than for RhoA and Cdc42 and that depletion of Rac1 only occurs upon massive down-regulation of RhoGDIα.

It has been reported that RhoGDIα knockdown in a variety of non-neuronal cells depletes total levels of Cdc42, RhoA and Rac1, but elevates active Cdc42 and Rac1 with no effect on active RhoA^30^. In contrast, we found that RhoGDIα knockdown in neurons decreased levels of both total and active forms of the three Rho GTPase proteins to a similar extent. This difference in findings could be due to the use of different experimental systems and to the longer period of our knockdown study (10 days compared to 3 days).

Although we observed that ATF4 knockdown leads to a significant reduction of RhoGDIα levels, ATF4 over-expression in neuronal cultures did not affect expression of this protein. This is consistent with our previous findings that ATF4 over-expression did not alter Rho GTPase protein family expression levels, or the densities of excitatory synapses or of mushroom spines^15^. Possible explanations for these observations are that ATF4 expression is already at maximal levels with respect to RhoGDIα regulation or that regulation of RhoGDIα requires ATF4 binding partners that are present in only limiting amounts.

Because ATF4 is a transcription factor, it is reasonable to expect its actions to originate at the level of gene regulation. However, in the case of Cdc42, ATF4 knockdown has no effect on mRNA levels, but leads to decreased stability of the protein^15^. The studies presented here show that RhoGDIα protein expression requires ATF4′s transcriptional activity. As evidence of this, wild-type, but not transcriptionally inactive ATF4 reversed the depletion of RhoGDIα protein levels caused by shATF4. Additionally, consistent with a transcriptional component to RhoGDIα regulation by ATF4, shATF4 caused a significant reduction in neuronal RhoGDIα mRNA levels. Our findings identify Arhgdia as a direct target for ATF4 regulation. We found that the Arhgdia 5′UTR contains a conserved consensus binding site for ATF4 and ChIP assays confirmed that ATF4 binds to this region of the Arhgdia promoter.

While our findings support Arhgdia as a potential direct target of ATF4, additional mechanisms may contribute to ATF4′s effects on RhoGDIα expression. Our time course data indicate that RhoGDIα protein expression begins to fall after ATF4 knockdown before there is significant reduction of RhoGDIα mRNA levels. One explanation for this might be the rapid regulation of miRNAs by ATF4 that in turn affect RhoGDIα translation. Recent studies have indicated that RhoGDIα expression is subject to downregulation by miRNAs such as mi483^49^ and mi151^50^. It remains to be seen whether these or other miRNAs targeting RhoGDIα are regulated by ATF4.

Rho GTPase proteins control a wide range of cellular activities including cell migration, proliferation, differentiation, adhesion, gene transcription, and cell cycle progression^51^,^52^. Moreover, deregulation of Rho GTPase pathways is associated with numerous disease states, such as neurodegenerative and developmental disorders^53^, tumorigenesis and cancer metastasis^54^,^55^,^56^. It is therefore significant that the ATF4-RhoGDIα-Cdc42 pathway is present not only in neurons, but also in other cell types such as melanocytes and kidney epithelial (NRK) cells. Both RhoGDIα and the Rho GTPses Cdc42, RhoA, and Rac1 have been implicated in regulation of cell migration^6^,^37^. As an indication of the physiologic relevance of ATF4 and of ATF4-RhoGDIα-RhoGTPase pathway in non-neuronal cells, we found that ATF4 knockdown significantly impaired migration and wound healing in NRK cultures. This phenotype can be associated with the significant reduction of Cdc42 protein we observed upon ATF4 down-regulation. However, based on our data, we can not exclude a contribution of the effect of ATF4 knockdown on RhoA and Rac1 expression to the observed deficits in cell migration.

ATF4 protein expression is regulated at both the transcriptional and translational levels as well as by efficient ubiquitination-dependent degradation^57^,^58^,^59^,^60^. Thus, there are multiple avenues and mechanisms by which cellular ATF4 levels may be modulated under both physiologic and pathologic conditions. This in turn has the potential to alter expression of RhoGDIα and therefore of Rho GTPase proteins in both neurons and non-neuronal cells and to have profound effects on their behavior.

## Methods

### DNA constructs

Lentiviral constructs were produced as previously described^15^. All the shRNAs were cloned in the pLVTHM vector (Addgene) using the following oligo DNA pairs:

Lenti-shRNA control:

5′-CGCGT**CACAGCCCTTCCACCTCCA**TTCAAGAGA**TGGAGGTGGAAGGGC**

**TGTG**TTTTTTA-3′ and

5′CGCGTAAAAAA**CACAGCCCTTCCACCTCCA**TCTCTTGAA**TGGAGGTGGA AGGGCTGTG**A-3′.

Lenti-shATF4:

5′-CGCGT**GCCTGACTCTGCTGCTTAT**TTCAAGAGA**ATAAGCAGCAGAGTC**

**AGGC**TTTTTTA-3′ and

5′-CGCGTAAAAAA**GCCTGACTCTGCTGCTTAT**TCTCTTGAA**ATAAGCAGCA**

**GAGTCAGGC**A-3′.

Lenti-shRhoGDIα:

5′-CGCGT**AGCACTCTGTGAACTACAA**TTCAAGAGA**TTGTAGTTCACAGAG TGCT**TTTTTTA-3′ and

5′-CGCGTAAAAAA**AGCACTCTGTGAACTACAA**TCTCTTGAA**TTGTAGTTCAC**

**AGAGTGCT**A-3′.

Lenti-shRhoGDIα#2:

CGCGT**GTCTAACCATGATGCCTTA**TTCAAGAGA**TAAGGCATCATGGTTAGACTTTTTTTA-3**′ and

5′-CGCGTAAAAAAA**GTCTAACCATGATGCCTTA**TCTCTTGAA**TAAGGCATCA**

**TGGTTAGAC**A-3′.

To overexpress ATF4 protein, rat ATF4 cDNA was cloned into the pWPI vector (Addgene) using the following PCR primer pair:

Lenti-ATF4:

5′-ATGACCGAGATGAGCTTCC-3′ and 5′- TTAAGGAACTCTCTTCTTC-3

Lenti-shATF4 addback was generated using the QuickChange Site-directed Mutagenesis kit (Stratagene). Point mutations were introduced into the Lenti-ATF4 at the recognition site for shATF4 (CCTGACTCTGCTGCTTAT to CCAGAGTCAGCTGCTTAC).

Lenti-shATF4 mut/addback was generated from shATF4addback by introducing point mutations at the DNA binding site (292RYRQKKR298 to 292GYLEAAA298).

### Lentiviral preparation

Lentiviral particles were produced using the 2^nd^ generation packaging system^15^. HEK293T cells were co-transfected with either lentiviral constructs for shRNA or overexpression and the packaging vectors psPAX2 and pMD2.G (Addgene) using calcium phosphate. 48 h and 72 h after transfection, supernatants were collected and lentiviral particles were concentrated 20–30x by centrifugation in Amicon Ultra centrifugal filters (100KD) (Millipore). Viruses were then aliquoted and stored at −80 °C. Viral titer ranged between 1–5 × 10^6^ virions/μl.

### Cell Culture and infection

#### Primary hippocampal neuronal cultures

Primary hippocampal and cortical cultures were prepared as previously described^15^. Briefly, hippocampi and cortices were dissected from E18 rat embryos, and, after dissociation, neurons were plated on poly-D-lysine-coated 12-well-plates at the density of 3 × 10^5^ cells/well. Neurons were maintained in Neurobasal medium (Invitrogen) supplemented with 2% B-27 (Invitrogen) and 0.5 mM glutamine (Invitrogen), and half of the medium was changed every 3 days. Neurons were infected with the aforementioned lentiviral-particles on *Day In Vitro* 5 (*DIV 5*) and protein extraction was performed 4, 8, and 14 days after infection.

#### Melan-a and NRK cell cultures

Melan-a cells (passage between 10 and 30) were cultured in RPMI-1640 medium (Invitrogen) supplemented with 10% fetal bovine serum (FBS, Hyclone), 200 nM 12-o-tetradecanoyl phorbol-13-acetate (TPA, Sigma-Aldrich), 1X Pen/Strep (Thermo Scientific), and 1 mM HCl. NRK cells (passage between 10 to 40) were cultured in DMEM (Invitrogen) supplemented with 10% bovine calf serum (CBS, Hyclone) and 1X Pen/Strep (Thermo Scientific).

Prior to infection cell lines were cultured on 6-well plates in medium with 10% serum. When 40% confluency was reached, the medium was replaced with one containing 2.5% serum. Cells were then infected twice (3 days apart) with the respective lentiviral particles and 4 μg/ml of hexadimethrine bromide (Polybrene), to enhance virus penetration into the cells. Medium was changed 24 h after infection to avoid Polybrene toxicity. Protein extraction was performed 10 days after the first infection.

#### Western immunoblotting

Western immunoblotting was performed on cultures at the indicated time points after lentiviral infection. Proteins were separated by electrophoresis 12% in Bis-Tris SDS-gels (Invitrogen) and transferred onto PVDF membranes. Membranes were cut to permit analysis of multiple proteins at the same time.

Membranes were blocked for 1 h at RT with 5% milk and then incubated overnight with primary antibody. The following primary antibodies were used: rabbit anti-ATF4 (1:500, PRF&L), rabbit anti-RhoGDIα (1:2000, Genentech), mouse anti-RhoA (1:1000; Cytoskeleton), mouse anti-RhoA-GTP (1:500; Santa Cruz), mouse anti-Rac1 (1:5000; Millipore), mouse anti-Cdc42 (1:500; BD), and mouse anti-GAPDH (1:2000; Imgenex) as loading control. Primary antibodies against ATF4, RhoA, Cdc42, Rac1, and GAPDH were previously validated^15^. Anti-RhoGDIα antibody detects a single protein (single band) located at the predicted molecular weight (28KDa), which is down-regulated by shRNA designed to specifically target RhoGDIα mRNA.

Proteins of interest were detected with horseradish peroxidase-conjugated secondary antibodies and captured on a film using enhanced chemiluminescence. When necessary several films were exposed for different time periods to obtain the proper balance between signal and background. Films were subsequently scanned and densitometric analysis of the bands was performed using the ImageJ program.

#### qRT-PCR

Total RNA was extracted from primary hippocampal and cortical cultures at the indicated time points after lentiviral infection, according to the RNeasy Mini Protocol (Quiagen kit). mRNA was then reverse-transcribed into cDNA using the 1^st^ Strand cDNA Synthesis System for quantitative RT-PCR (Origene) following the manufacturer’s instructions. Reaction mixtures were diluted 5-fold and subjected to qRT-PCR amplification (Eppendorf). The following primers were used:

ATF4: F 5′-ATGCCAGATGAG CTCTTGACCAC-3′ and R 5′-GTCATTGTCAGAGGGAGTGTCTTC-3′; RhoGDIα: F 5′-TGTGCTGCTGTTGCTTCC-3′ and R 5′-GCTCGGCTGGCTTTGT-3′; UCHL1: F 5′-CCAAGTGTTTCGAGAAGAACG-3′ and R 5′ GCTAAAGCTGCAAACCAAGG-3′; Smurf1: F 5′-AGTTCGTGGCCAAATAGTGGTC-3 and R 5′-GTTCCTTCGTTCTCCAGCAGTC-3; UBE3A: F 5′-ATGTGGAAGCCGGAATCTCG-3′ and R 5′-CCCAATGAAGAAGGGAGGCA-3′; αTubulin: F 5′-TACACCATTGGCAAGGAGAT-3′ and R 5′-GGCTGGGTAAAT GGAGAACT-3′. αTubulin was used as an internal control for normalization.

#### CDC42-GTP, RAC1-GTP Pull-Down

To analyze the GTP-bound forms of Cdc42 and Rac1 (active forms), pull-down experiments were performed on infected hippocampal neurons. Briefly, cells were lysed in RIPA buffer and centrifuged at 15,000 rpm for 10 min at 4 °C. Supernatants were mixed with PAK-GST beads which binds specifically to GTP-bound, and not GDP-bound, Rac and Cdc42 proteins (Cytoskeleton). Levels of Rac1-GTP and Cdc42-GTP were detected by Western immunoblotting using anti-Rac1 and anti-Cdc42 antibodies described before.

#### Chromatin Immunoprecipitation (ChIP)

ChIP assays were performed using the Simple ChIP Plus Enzymatic Chromatin IP Kit (Cell Signaling) according to the manufacturer’s instructions. Briefly, rat primary cortical neurons were cultured in 150-mm dishes for 2 weeks. Formaldehyde was added (final concentration 1%) to cross-link chromatin and quenched 10 min later by adding glycine. Nuclei were isolated and subjected to chromatin digestion by micrococcal nuclease at 37 °C for 20 min, followed by brief sonication. Lysates were clarified by centrifugation at 10,000 rpm for 10 min at 4 °C. Extracts were then incubated with ATF4 antibody or normal rabbit IgG overnight at 4 °C with rotation. Chromatin-antibody complexes were captured by incubation with protein G magnetic beads. The DNA fragments were released by incubation with NaCl at 65 °C for 3 h. Purified DNA was subjected to real-time quantitative PCR. To cover the predicted ATF4 binding site in the RhoGDIα promoter region, the following primers were used for qPCR, f1: GCGAGAGCGGAAGTCTTGTGAC, r1: TCGCCGCACAAAGCCAACCCAC, r2: CCACTCACCGGAGGCTCGAC.

#### Wound-healing (Scratch) assay

NRK cells were plated into six-well plates in culture medium. Cells were infected twice with the respective lentiviral construct. The confluent cell monolayer (usually 10 days after the first infection) was scratched in a straight line with a sterile 20 μL pipette tip to create a cell-free gap and then washed with PBS followed by addition of fresh medium. The wound was imaged in the same area at 20X magnification at 1 h, 7 h and 24 h after the scratch. Wound width was measured using Image J software (NIH, Rockville, MD, USA). Briefly, the boundary of the cells on each side of the scratch was assessed and represented by a vertical line. The width between both boundaries was then measured and considered to be the width of the scratch.

#### Statistical analysis

Data are shown as means ± sem. Comparison between two groups was performed with the two-tailed paired Student’s t-test. Statistical significance was considered for p < 0.05.

## Additional Information

**How to cite this article**: Pasini, S. *et al*. Activating Transcription Factor 4 (ATF4) modulates Rho GTPase levels and function via regulation of RhoGDIα. *Sci. Rep*. **6**, 36952; doi: 10.1038/srep36952 (2016).

**Publisher’s note**: Springer Nature remains neutral with regard to jurisdictional claims in published maps and institutional affiliations.

## Supplementary Material

Supplementary Information
