# Supplementary material for: Activating Transcription Factor 4 (ATF4) modulates Rho GTPase levels and function via regulation of RhoGDIα
Source: Sci Rep. 2016 Nov 14;6:36952. doi: 10.1038/srep36952 (PMC5107905; doi:10.1038/srep36952)

Activating Transcription Factor 4 (ATF4) modulates Rho GTPase levels and function via regulation of RhoGDIα.

Silvia Pasini, Jin Liu, Carlo Corona, Eugenie Peze-Heidsieck, Michael Shelanski, and Lloyd A. Greene.

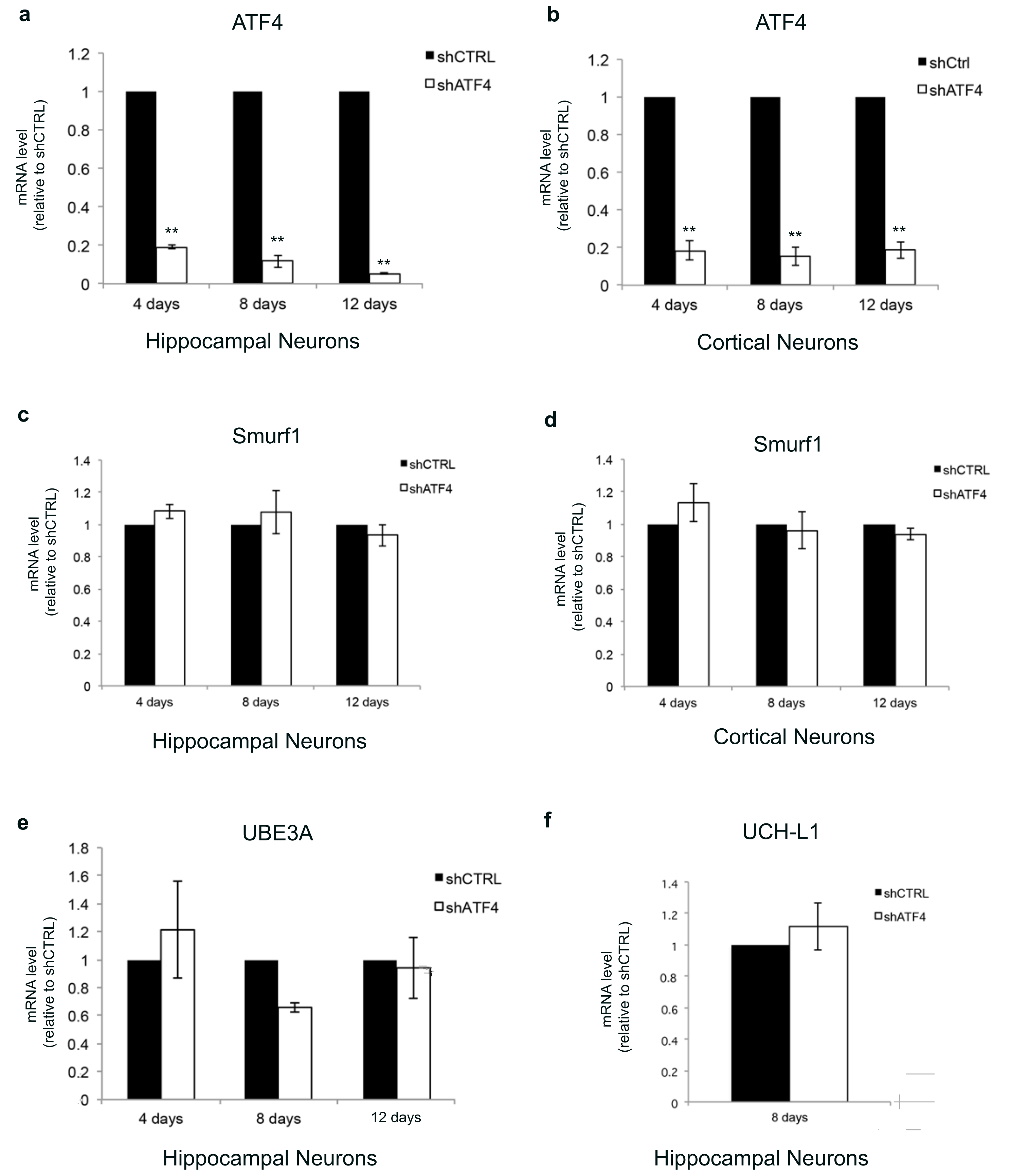

Supplementary Figure 1. ATF4 knockdown in primary hippocampal and cortical neurons does not alter mRNA levels of the E3 ubiquitin ligases Smurf1, UBE3A, and UCH-L1. Total RNA from cultured hippocampal and cortical neurons was extracted at the indicated time points and subjected to quantitative real time PCR to detect message levels for ATF4 (**a**, hippocampal neurons) (**b**, cortical neurons); Smurf1 (**c**, hippocampal neurons) (**d**, cortical neurons); UBE3A (**e**, hippocampal neurons), and UCH-L1(**f**, hippocampal neurons). Data are expressed as mean ± sem of 3 independent experiments.

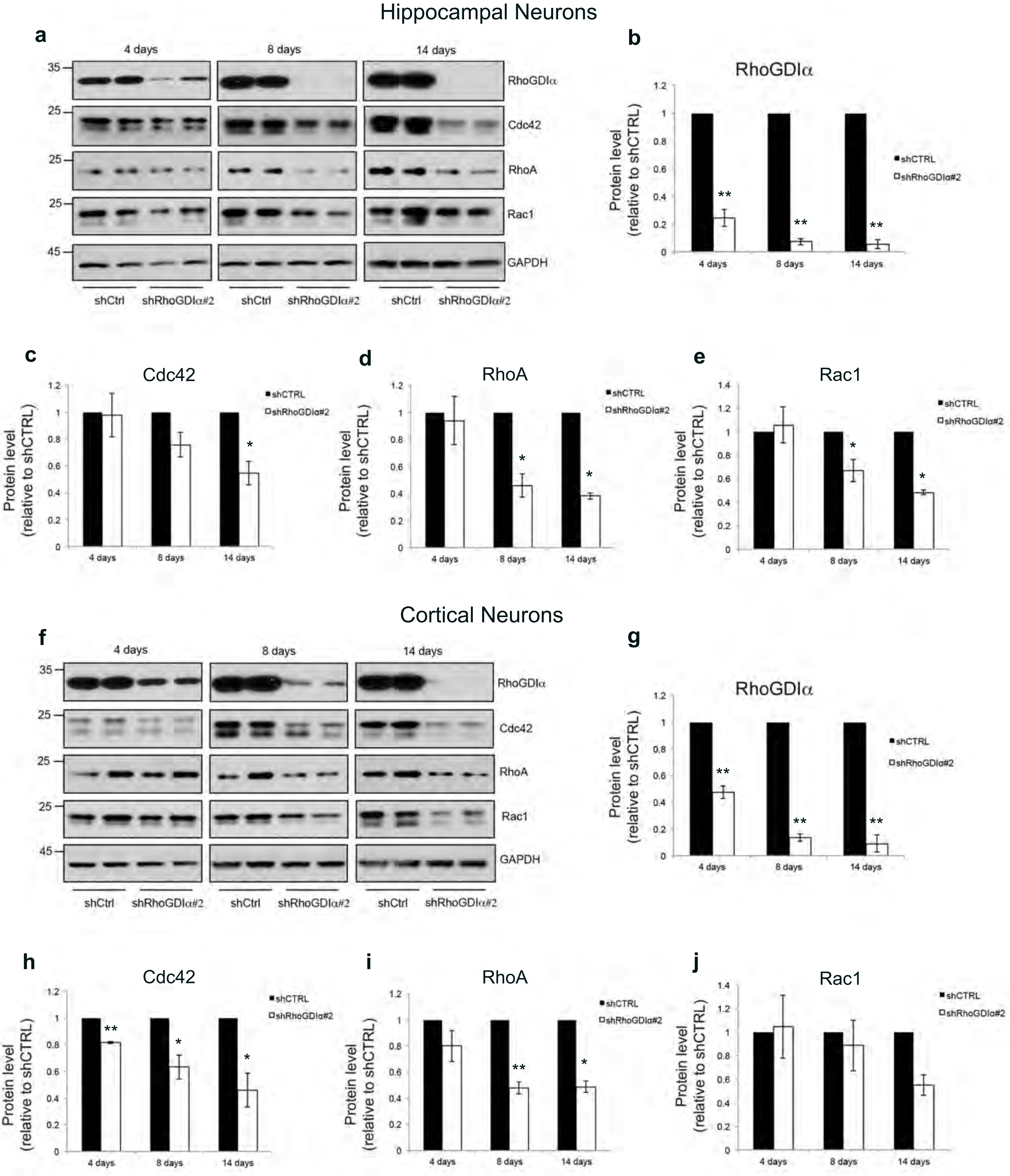

Supplementary Figure 2. RhoGDIα knockdown in cultures of hippocampal and cortical neurons using a second shRNA sequence (shRhoGDIα#2) reduces the total forms of the Rho GTPases Cdc42, RhoA, and Rac1. (**a**) Representative Western immunoblot showing a time course of the effect of RhoGDIα knockdown on Rho GTPase family protein levels. Primary hippocampal neurons were infected with lenti-shCTRL or lenti-shRhoGDIα#2 at 5 DIV and total cell lysates were collected at the indicated time points after infection and analyzed by Western immunoblotting. (**b-e**) Quantification of RhoGDIα **b**), Cdc42 (**c**), RhoA (**d**), and Rac1 (**e**) protein levels at the indicated time points relative to control. (**f**) Representative Western blotting showing a time course of the effect of RhoGDIα knockdown on Rho GTPase family protein levels. Primary cortical neurons were infected with lenti-shCTRL or lenti-shRhoGDIα#2 at 5 DIV and total cell lysates were collected at different time points after infection and analyzed by Western immunoblotting. (**g-j**) Quantification of RhoGDIα (**g**), Cdc42 (**h**), RhoA (**i**), and Rac1 (**j**) protein levels at the indicated time points relative to control. Data are expressed as mean ± sem of independent experiments (4 days n=5, 8 days n=5, 14 days n=3) (*p<0.05, **p<0.001). Full size Westen immunoblots are shown in Suppl. Figure 8.

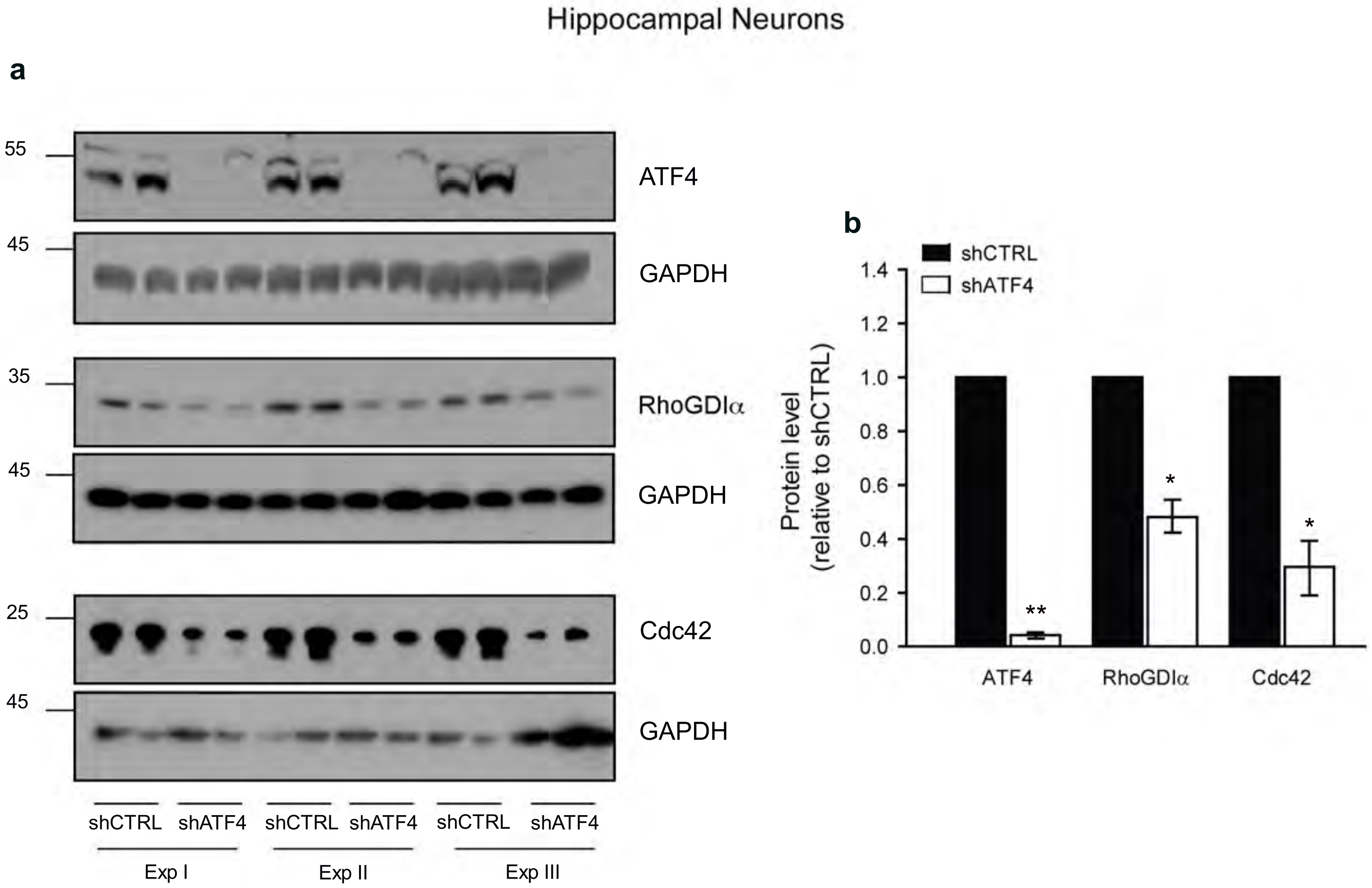

Supplementary Figure 3. ATF4 knockdown in cultures of hippocampal neurons reduces RhoGDIa and Cdc42 protein. (**a**) Representative Western blots showing the effect of ATF4 knockdown on RhoGDIa and Cdc42 proteins level in cultured hippocampal neurons. Cultured hippocampal neurons were infected at 5 DIV with lenti-shCTRL or lenti-shATF4 and total cell lysates were collected 14 days after the infection and analyzed by Western immunoblotting. Full size Westen immunoblots are shown in Suppl. Figure 8. (**b**) Quantification of ATF4, RhoGDIa, and Cdc42 protein levels 14 days of ATF4 downregulation relative to control. Data are expressed as mean ± sem of 3 independent experiments (*$p<0.05$, **$p<0.001$).

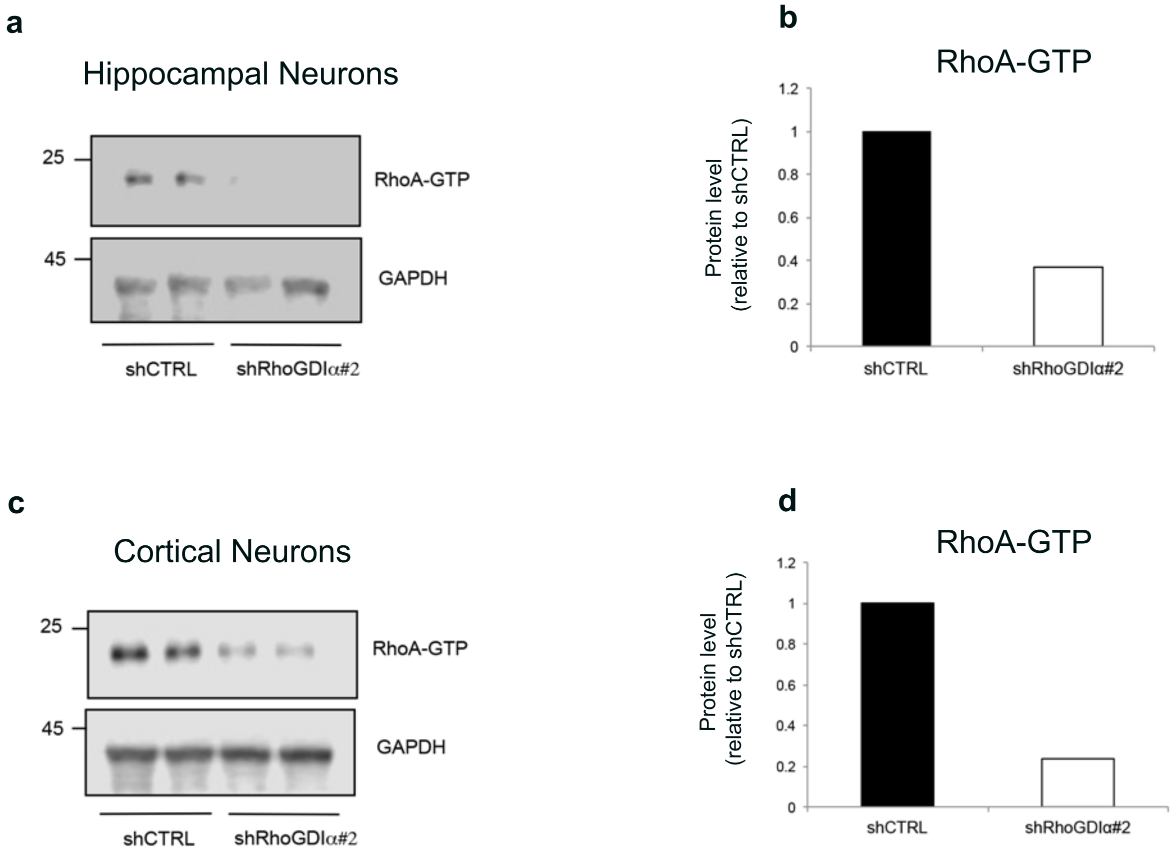

Supplementary Figure 4. RhoGDIα knockdown in cultures of hippocampal and cortical neurons using a second shRNA sequence (shRhoGDIα#2) reduces the active form of RhoA. (**a,c**) Representative Western blots showing the effect of RhoGDIα knockdown on RhoA-GTP protein level in cultured hippocampal (**a**) and cortical (**c**) neurons. Cultured hippocampal and cortical neurons were infected at 5 DIV with lenti-shCTRL or lenti-shRhoGDIα#2 and total cell lysates were collected 10 days after the infection and analyzed by Western immunoblotting using an antibody that binds specifically to RhoA-GTP. (**b,d**) Quantification of RhoA-GTP relative to control after RhoGDIα knockdown in cultures of hippocampal (**b**) and cortical (**d**) neurons. Data are expressed as mean of 1 experiment run in duplicate. Full size Western immunoblots are shown in Suppl. Figure 8.

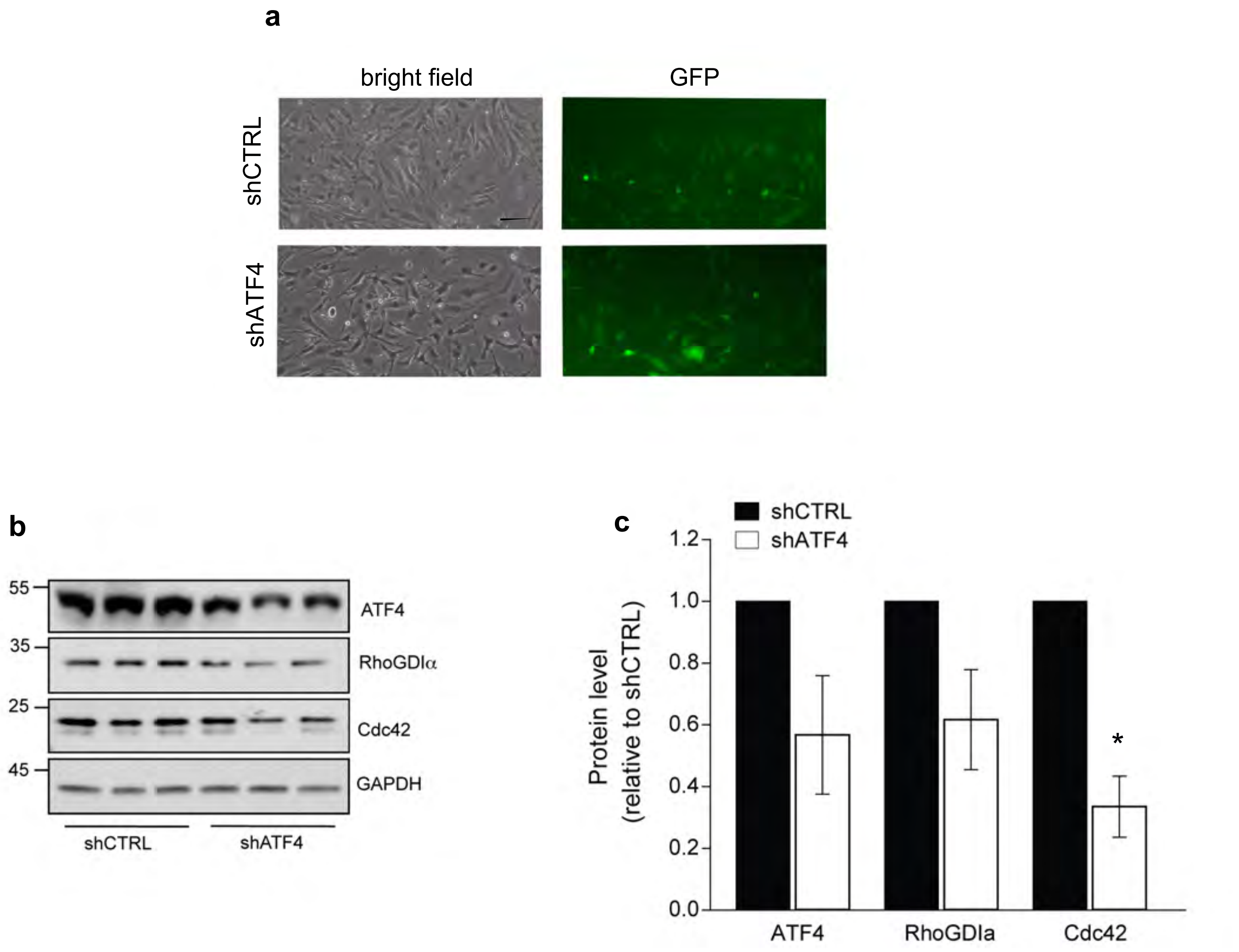

Supplementary Figure 5. The ATF4 - RhoGDIα - Cdc42 pathway in Melan-a cells.
(**a**) Representative images of Melan-a cells subjected to a double infection (3 days apart) with either lenti-shCTRL or lenti-shATF4. Images were taken in both bright field (total cells) and a green channel (infected cells) 10 days after the first infection. Scale bar 50 µm.(**b**) Representative Western immunoblots showing the effect of ATF4 knockdown on RhoGDIα and Ccd42 protein levels in Melan-a cells. Melan-a cells were subjected to double infection (3 days apart) with either lenti-shCTRL or lenti-shATF4. Total protein lysates were collected 10 days after the first infection and subjected to Western immunoblotting. Full size Western immunoblots are shown in Suppl. Figure 8. (**c**) Quantification of ATF4, RhoGDIα, and Cdc42 protein levels in shATF4-infected cells compared to shCTRL-infected cells. Data are expressed as mean ± sem of 3 independent experiments.(*$p<0.05$).

Supplementary Figure 6

Westen immunoblots Figure 1a

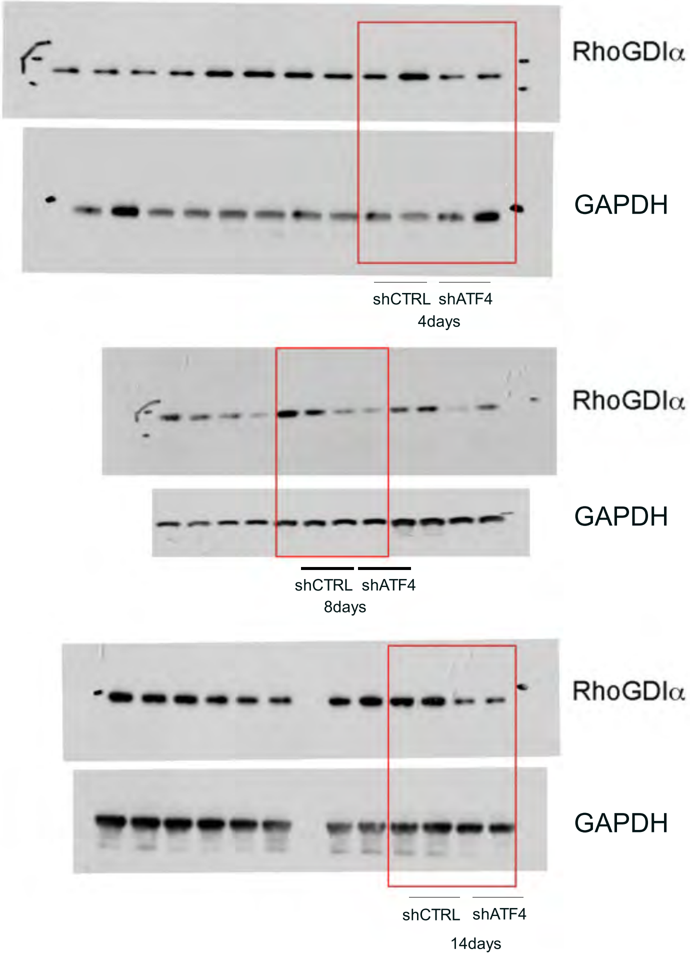

Westen immunoblots Figure 1e

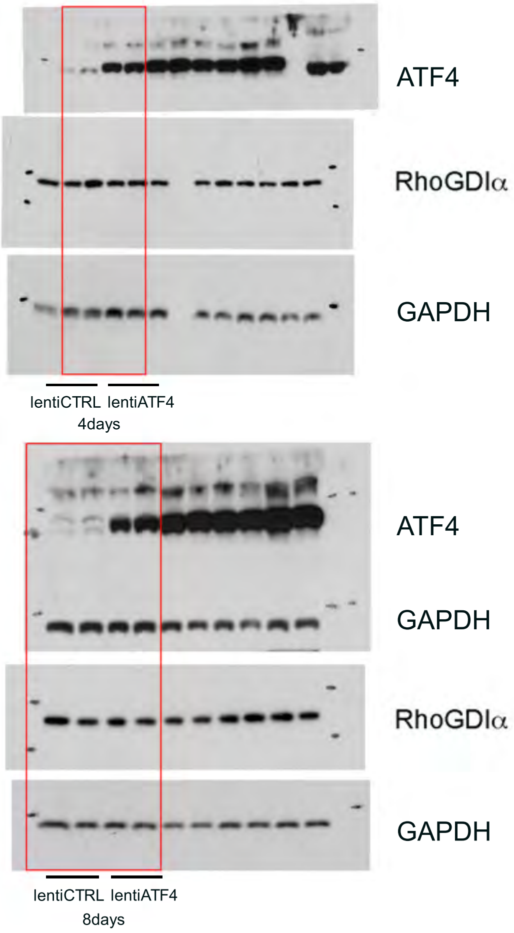

Westen immunoblots Figure 1c

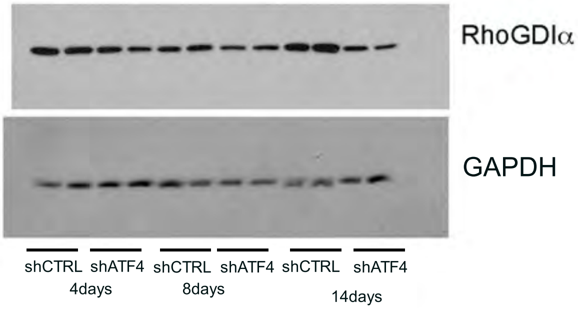

Westen immunoblots Figure 1g

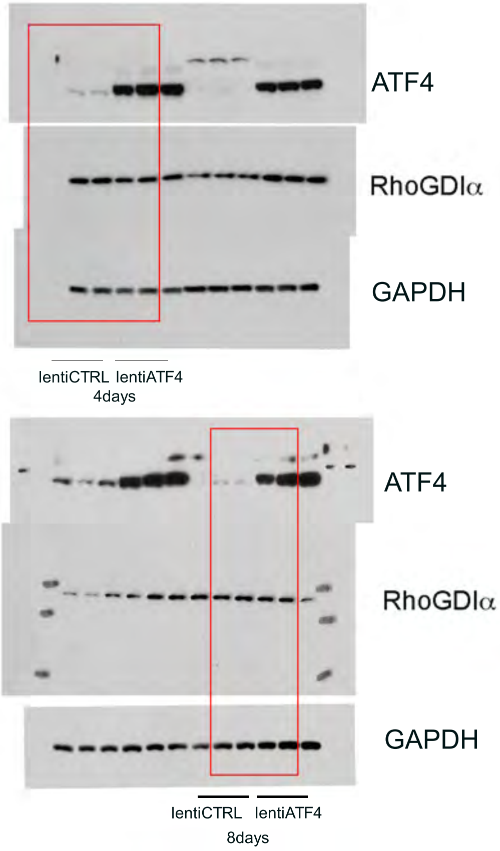

Westen immunoblots Figure 6

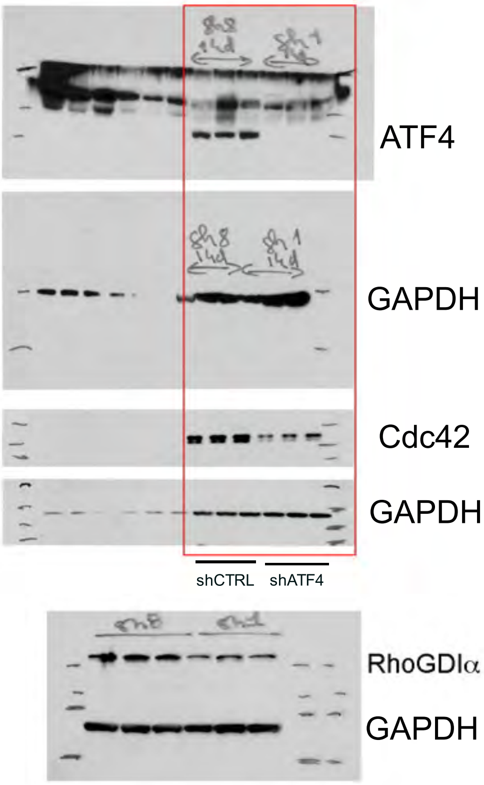

Supplementary Figure 7

Westen immunoblots Figure 2

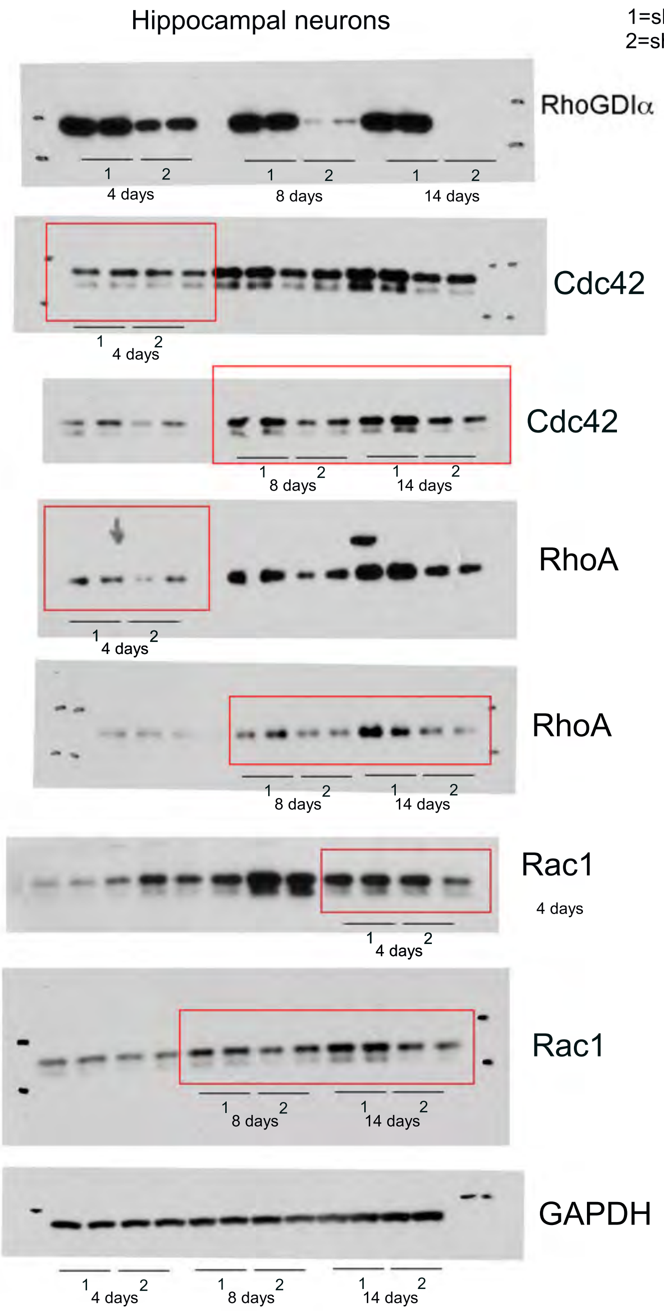

1=shCTRL
2=shRhoGDIα

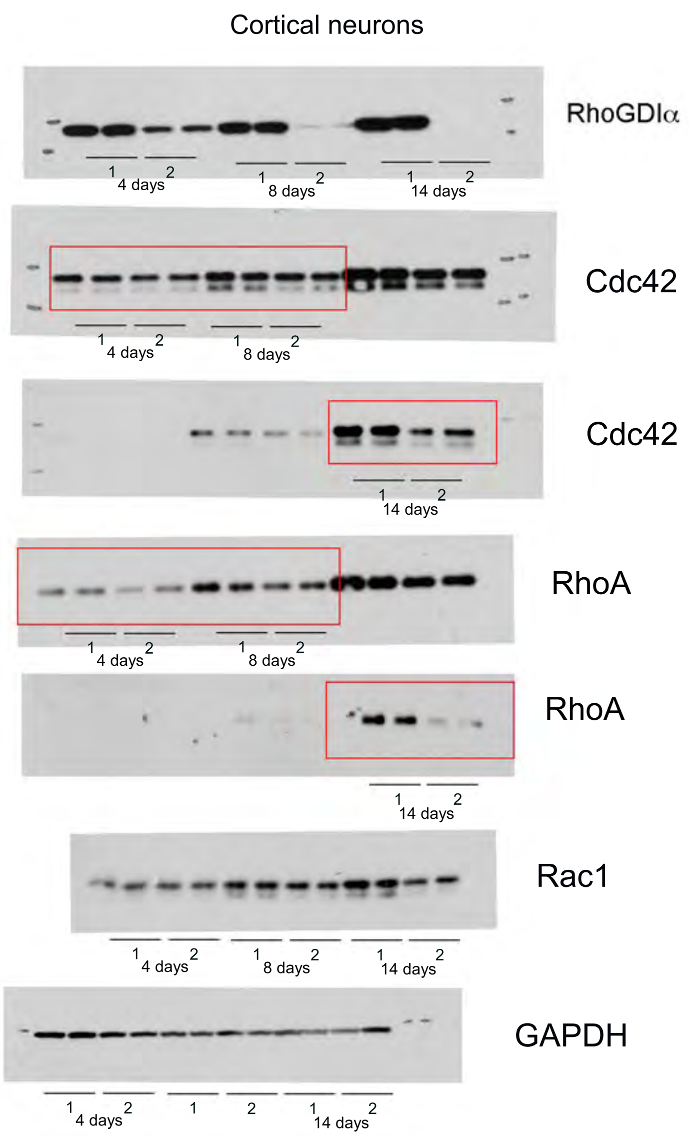

Westen immunoblots Figure 4

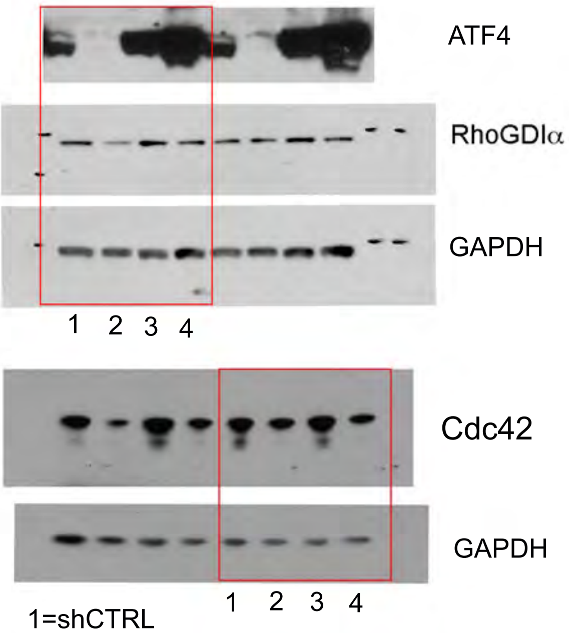

1=shCTRL
2=shATF4
3=shATF4+ATF4add
4=shATF4+mutATF4add

Westen immunoblots Fig. 3

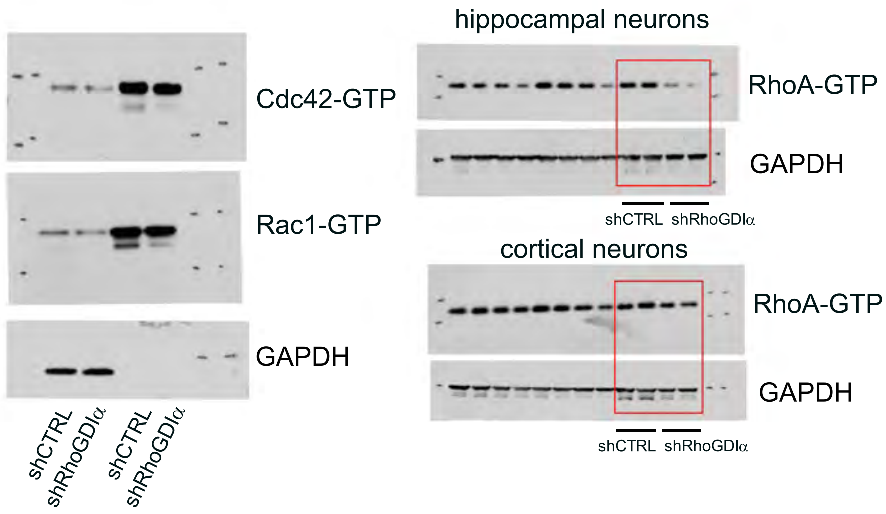

Supplementary Figure 8

Western immunoblots Suppl. Figure 2

1=shCTRL
2=shRhoGDIα#2

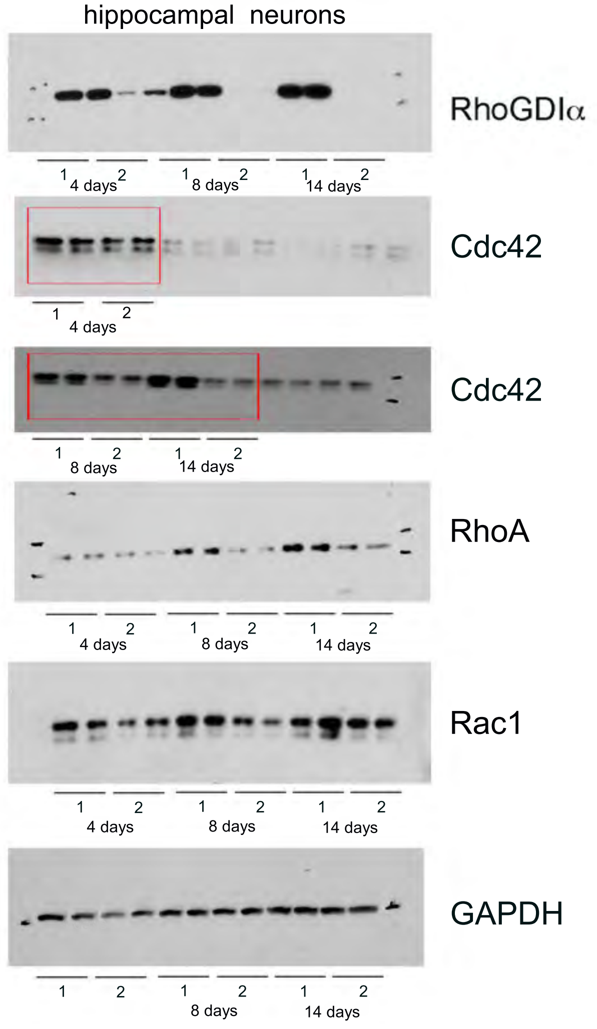

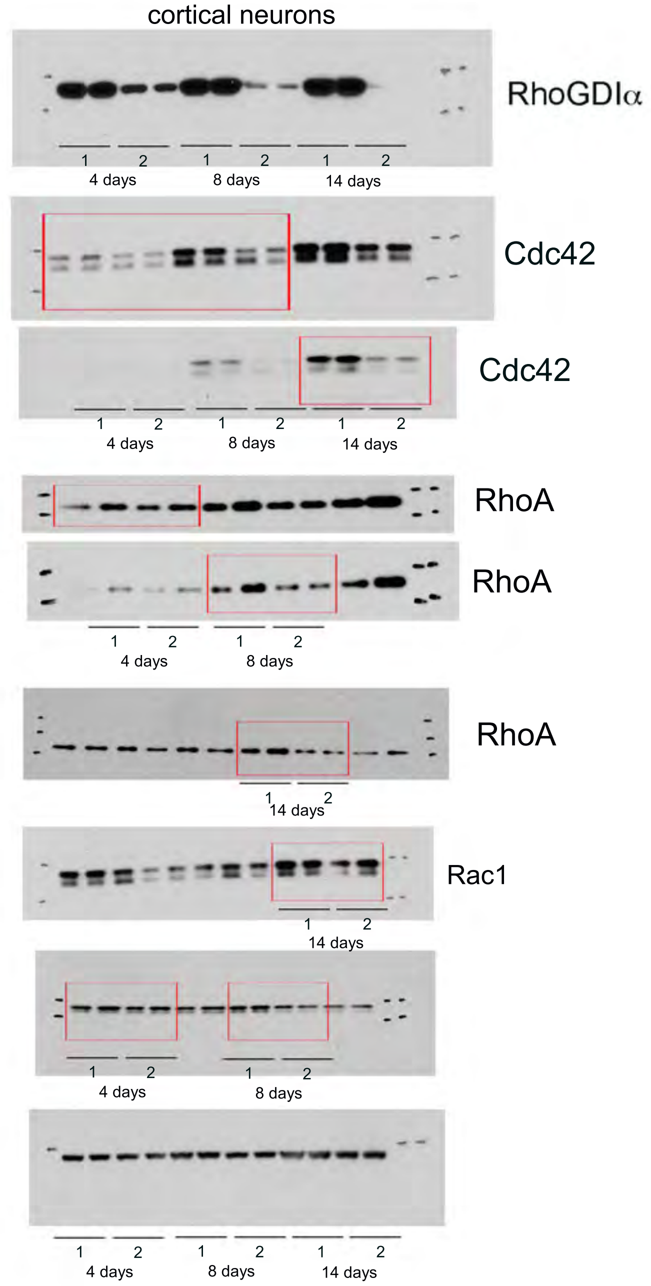

Western immunoblots Suppl. Figure 3

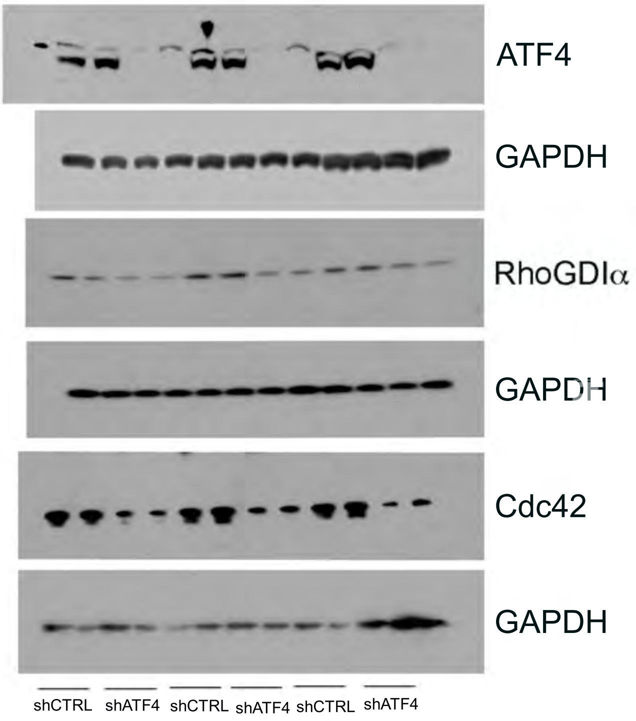

Western immunoblots Suppl. Figure 4

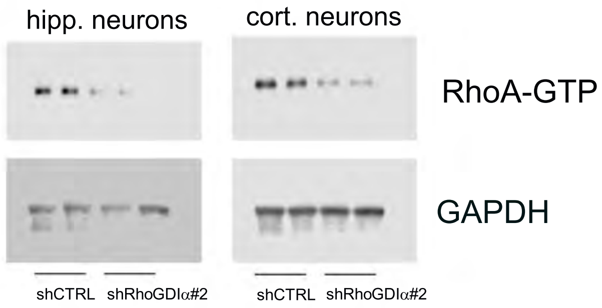

Westen immunoblots Suppl. Figure 5

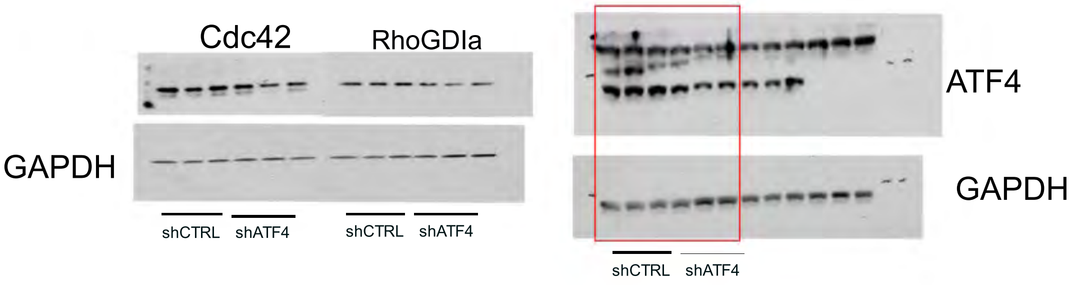

Supplement: Supplementary Information [file srep36952-s1.pdf]
